# Biotechnology: a non-existing word/world for the Brazilian deaf community

**DOI:** 10.1186/1753-6561-8-S4-P262

**Published:** 2014-10-01

**Authors:** Ruth Mariani, Cristina Maria Delou, Andressa da Silva Souza, Valentinne Rodrigues Valentim, Deivid dos Passos, Andreza Rosa, Larissa de Oliveira Farias, Karoline Farias Marrocos Moraes, Helena Carla Castro

**Affiliations:** 1 Programa de Pós-Graduação em Ciências e Biotecnologia, Instituto de Biologia, Universidade Federal Fluminense, RJ, Brazil; 2 Laboratório de Antibióticos, Bioquímica, Ensino e Modelagem Molecular, Instituto de Biologia, Universidade Federal Fluminense, RJ, Brazil

## Background

Biotechnology is a complex theme that is directly or indirectly present in our lives, from food to medicine. Despite that, the understanding about biotechnology is still difficult for most of society due to this complexity. This is worsened for Brazilian deaf community since the word *Biotechnology *does not exist in Brazilian Sign Language (LIBRAS), the official deaf language. This young language, similar to some foreigner Sign languages, still misses several scientific terms/words [1]. These terms have to be created by the deaf community to help them guarantee the information access, their citizens rights and a better academic formation [1,2]. Therefore, our purpose as a group formed by deaf and hearing people is to suggest a sign for the word *Biotechnology *to help on the understanding of the biotechnological area.

## Methods

Initially six deaf students from a public high school were invited to participate in this work as requested by the deaf community for this type of project. To guarantee their understanding, we created a class about this theme in an inquiry-oriented teaching way, also using materials for contextualization (*e.g*. bottle of transgenic vegetal oil) [1,3].

## Results and conclusions

LIBRAS is a language whose signs represent the concept of the words [2,4]. Therefore the word should be previously fully understood by deaf before creating the respective sign. The six deaf students that participated in this work are from a previous stimulated environment, the project *Spreadthesign *(http://www.spreadthesign.com), an international dictionary of 25 different sign languages from several countries now including LIBRAS from Brazil with our participation. We were also helped by an interpreter that always interrupted the class when one concept seemed to be not fully understood. Thus, cheese production, the snake venom biotechnological potential, the creation of transgenics for food production, and the insulin biotechnological production were fully discussed in this class. In the end, three signs for *Biotechnology *emerged: a) the combination of the sign of *biology *- right hand signing the letter B spinning around slightly in the air - with another movement; so the final sign is the right hand in the position of letter B (for Bio) rubbed in the palm of the left hand, to remind the process of generating some product using biotechnological methods; b) They used dactylology for the letters B, I and O and the sign of technology (hands in the position of letter T slightly spinning in front of each other). Finally, c) the combination of the sign meaning *life *- right hand with fingers together on the left shoulder - for Bio, followed by the sign of technology described above. After those sign propositions construction, they decided to send them to the Brazilian Biotechnology Society Event (5^th ^SBBiotec) to ask for the society analysis and oppinion. As citizens, deaf community should be allowed to understand the implications of the major changes brought about by biotechnological advances [3-5] and on that matter, the creation of this and other scientific signs may help this community on guarantee the access and understand of current biotechnological information.
